# Comprehensive Analysis of HOX Family Members as Novel Diagnostic and Prognostic Markers for Hepatocellular Carcinoma

**DOI:** 10.1155/2022/5758601

**Published:** 2022-02-23

**Authors:** Zhipeng Jin, Dongxu Sun, Mengying Song, Wenjing Zhu, Huayuan Liu, Jianping Wang, Guangjun Shi

**Affiliations:** ^1^Department of Hepatobiliary Surgery, Qingdao Municipal Hospital, Qingdao, China; ^2^Department of Operation Room, Cancer Hospital of China Medical University, Shenyang, China; ^3^Clinical Research Center, Qingdao Municipal Hospital, Qingdao, China

## Abstract

**Background:**

The homeobox (HOX) gene family has been found to be involved in human cancers. However, its involvement in hepatocellular carcinoma (HCC) has not been well documented. Here, we comprehensively evaluated the role of HOXs in HCC.

**Methods:**

RNA sequencing profile of TCGA-LIHC and LIRI-JP were obtained from the Cancer Genome Atlas (TCGA) and the International Cancer Genome Consortium (ICGC), respectively. Data of TCGA-LIHC methylation were downloaded from UCSC Xena. Genetic alteration data for the TCGA samples was obtained from cBioPortal and GSCA. The diagnostic efficiency was assessed using ROC curves. The prognostic significance was evaluated by the Kaplan–Meier method and Cox regression analysis. Subsequent functional analysis was performed through the clusterProfiler package. ssGSEA, ESTIMATE, and TIDE algorithms were employed to explore the relationship between HOXs and the HCC microenvironment. Finally, pRRophetic package and NCI-60 cancerous cell lines were applied to estimate anticancer drug sensitivity.

**Results:**

The mRNA levels of HOXs in HCC tissues were higher than those of noncancerous tissues and were correlated with poor overall survival (OS). HOXA6, C6, D9, D10, and D13 could serve as independent risk factors for OS. Further functional analysis revealed that these five HOXs regulate the cell proliferation, cell cycle, immune response, and microenvironment composition of HCC. In addition, the aberrant expression and methylation of HOXs is of great value in the diagnosis of HCC.

**Conclusion:**

HOXs play crucial roles in HCC and could serve as potential markers for HCC diagnosis and prognosis.

## 1. Introduction

Hepatocellular carcinoma is an important cause of human cancer-related deaths worldwide, and its incidence continues to rise [1]. Meanwhile, it is also one of the cancers with the worst prognosis. According to statistics, the median survival time of advanced cases is only 2-3 years [2]. Surgery is the most important treatment for early HCC. However, due to the lack of specific symptoms, patients miss the best opportunity for surgery [1]. Posthepatic resection recurrence is another considerable challenge. Even in patients with early HCC, the 5-year recurrence rate was close to 70% [3]. The high recurrence rate and poor curative effect are related to the complicated pathogenesis of HCC, as various networks of molecules and signaling pathways are involved in its occurrence and development [4]. Therefore, the discovery of new molecules involved in HCC progression and the identification of new diagnostic markers and therapeutic targets is critically important for improving HCC patients' prognosis.

The HOX genes share a DNA sequence called “homeobox” which consists of a 120-base pair and encodes a polypeptide consisting of 61 amino acids, known as a homeodomain [5]. For the first time, HOX genes were found to be involved in the embryogenesis of *Drosophila melanogaster* [6]. Notably, structural and developmental variations were observed in mutant *D. melanogaster*, such as the replacement of antennae with legs. These anomalies, caused by mutations, are called “homeotic” transformations. In 1921, geneticists proposed the existence of genes that specifically regulate these transformations [7]. Seventy years later, the protein structures of such genes were identified in humans, and termed “homeotic” or “HOX” genes [8, 9]. The discovery of the HOX gene is crucial to understanding the genetic control mechanisms of embryonic development. In mammals, diverse HOX members control the development of different parts of the body [10].

Thirty-nine HOXs were identified in the human genome, located on chromosomes 2, 7, 12, and 17, and divided it them into four gene clusters (A, B, C, and D, respectively). Each cluster was also divided into 13 paralog groups. Each gene cluster contains from 9 to 11 members (Figure S1) [11]. Over the past century, many studies found that HOXs were closely related to human cancers [12–15]. Meanwhile, the aberrant methylation of HOX genes was also identified as a characteristic of cancers [16]. All these findings have shown the potential of HOXs as biomarkers for human cancers.

In HCC, the abnormal expression of few HOX members was established to be involved in disease progression [17, 18]. However, the significance of most HOX members is still not clear. Here, we integrally analyzed the genomic data of HOXs in HCC, and assessed their diagnostic and prognostic value.

## 2. Materials and Methods

**Figure 1:**
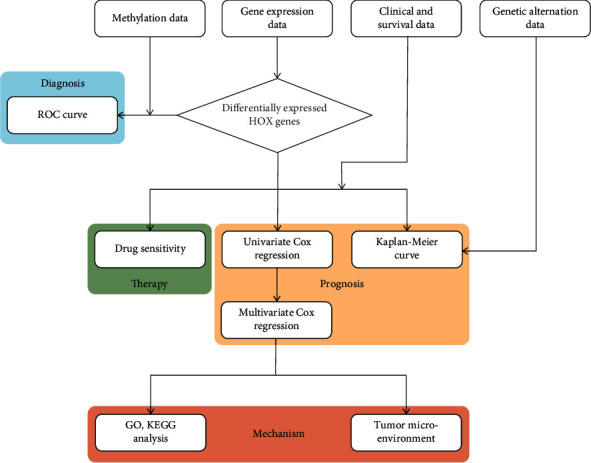
Flowchart of the present research.

The flowchart of this study is presented in Figure 1.

### 2.1. Datasets Sources and Processing

The RNA-seq (FPKM format) and clinical data of TCGA-LIHC were downloaded from the GDC Data Portal (https://portal.gdc.cancer.gov/). The RNA-seq (FPKM) of LIRI-JP was downloaded from ICGC Data Portal (https://dcc.icgc.org/). The limma package [19] in R software was applied to identify HOX genes differentially expressed between cancerous tissues and adjacent noncancerous tissues. The threshold was set as |log2 Fold Change| >1.5 and a *P* value <0.05. The methylation data for TCGA-LIHC was downloaded from the University of California, Santa Cruz (UCSC; Santa Cruz, CA, USA) Xena data portal (https://xena.ucsc.edu/). The beta values from the same sample but from different vials/portions/analytes/aliquotes were averaged, whereas the beta values from different samples were combined into a genomic matrix. The Corrplot [20] package in R software was used to evaluate the correlation between the gene expression or the methylation levels of the CpG sites and the corresponding gene expression.

### 2.2. Assessment of Genetic Alterations in HOX Genes

cBioPortal is an interactive open-source platform that provides large scale cancer genomics data sets (https://www.cBioPortal.org/) [21]. We obtained the genomic data of samples in TCGA-LIHC (Firehose Legacy), including mutations, putative copy-number alterations from GISTIC, and mRNA expression z-Scores (RNASeq V2 RSEM) with a z-score threshold ± 2.0. All samples were divided into two groups. The altered group included 59 samples with mutations or CNA, whereas the unaltered group consisted of 301 samples without mutations or CNA. Then, the differences in the overall survival and disease-free statuses (since initial treatment) between the two groups were analyzed using K–M survival analysis.

Gene Set Cancer Analysis (GSCA) is an integrated database for the analysis of cancer genomics (http://bioinfo.life.hust.edu.cn/GSCA/#/) [22]. We obtained the details of the SNV and CNV of HOX family genes in TCGA-LIHC from the GSCA database. The mutation data showed seven types of deleterious mutations. The CNV data were processed through GISTICS2.0. Based on the GISTIC score, CNV was divided into four categories.

### 2.3. PPI Network and Functional Enrichment Analysis

The genes coexpressed with HOX genes in TCGA-LIHC were collected from the UALCAN database (https://ualcan.path.uab.edu/) [23]. A correlation coefficient ≥0.4 was considered to indicate a significant correlation. Based on genes significantly correlated with HOXs, we constructed a PPI network using STRING v.10.0b (https://string-db.org/) [24]. Next, we screened the hub genes and visualized the STRING results using Cytoscape v3.8.0 (https://cytoscape.org/cy3.html) [25]. Then, the clusterProfiler package [26] in R was employed to identify the Gene Ontology (GO) terms (including cellular component, biological process, and molecular function) and Kyoto Encyclopedia of Genes and Genomes (KEGG) pathways that were enriched by hub genes and to visualize the results.

### 2.4. Relationship between HOXs and HCC Microenvironment

The abundance of 24 immune cell types was predicted by calculating the single-sample gene set enrichment analysis (ssGSEA) scores based on the gene set signatures of each type of the immune cells through ImmuCellAI (http://bioinfo.life.hust.edu.cn/ImmuCellAI/#!/) [27]. Further, we obtained bubble plots presenting the correlation between the mRNA expression of the HOX genes and the estimated abundance of immune cells from the GSCA database. Then, the ssGSEA scores of 13 immune functions of each HCC sample were quantified using the GSVA package [28] in R software.

Afterwards, the stromal cell levels in HCC tissues were estimated using the ESTIMATE algorithm, which analyzes the composition of the microenvironment and calculates the tumor purity based on the gene expression data [29].

### 2.5. Role of HOXC6 in Immune-Checkpoint Inhibitor Treatment

First, the correlation of HOXC6 with two types of immune-checkpoint inhibitor treatment response-related biomarkers, immune-checkpoint genes, and tumor mutation burden (TMB) was evaluated. Gene expression and somatic mutation data were obtained from TCGA-LIHC, and TMB was calculated based on the somatic mutation data collected.

Then, the Tumor Immune Dysfunction and Exclusion (TIDE) algorithm (http://tide.dfci.harvard.edu/login/) [30] was applied to predict the response to anti-PD-1 and anti-CTLA4 treatment.

### 2.6. Anticancer Drug Sensitivity Prediction

We analyzed the relationship between HOX genes expression and anticancer drugs sensitivity by estimating the half-maximal inhibitory concentration (IC50). The IC50 of sorafenib in each HCC sample was predicted using the pRRophetic package [31] in R.

The NCI-60 human cancer cell line panel [32] was previously used by cancer investigators and the NCI Developmental Therapeutics Program (DTP) to discover novel anticancer drugs [33]. We obtained data of the RNA-seq/composite expression and the compound activity (average *z* score) of DTP NCI-60 from CellMiner v2.6 (https://discover.nci.nih.gov/cellminer/home.do) [34].

### 2.7. Statistical Analysis

Statistical analyses were conducted in R software (version 4.0.2). Wilcoxon rank-sum test was used to analyze the differences between the two subgroups. Then, the Kaplan–Meier method and the log-rank test were utilized to analyze the differences in the survival between the groups of patients. The best cut-off values of the groups were determined using the survminer package in R. Moreover, independent prognostic analysis was conducted via Cox proportional hazards models. We factored gender, age, tumor stage, and tumor differentiation into confounding factors and excluded patients with multiple sets of expression data, missing expression data, or without the aforementioned clinical information. Pearson correlation test was employed to measure the correlation between variables. Receiver operating characteristic curves were established to evaluate the diagnostic values of HOXs, and the pROC package was used to quantify the area under the curve (AUC). In all statistical analyses, a *P* value <0.05 was considered statistically significant.

## 3. Results

### 3.1. Increased HOX Family Genes Expression in HCC

**Figure 2:**
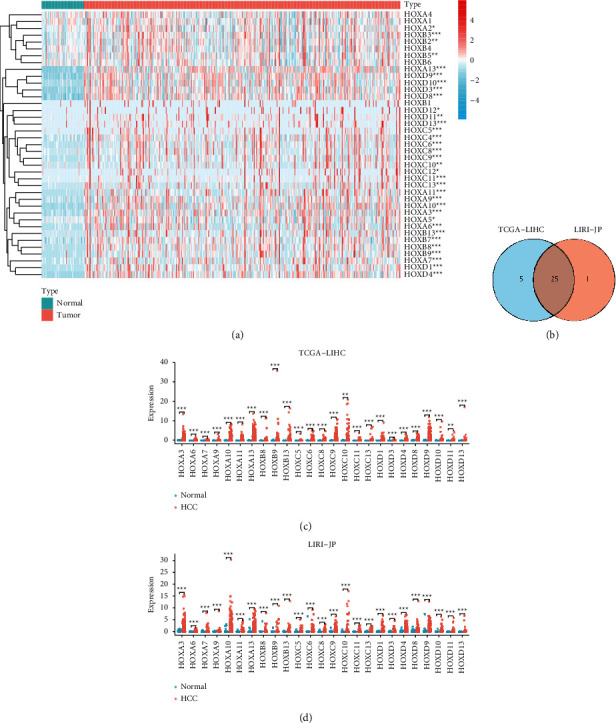
Expression profile of HOXs in HCC. (a) Heatmap of HOX family genes expression in TCGA-LIHC; (b) venn diagram of differentially expressed HOX genes in two HCC cohorts; (c) expression of 25 DEGs in TCGA-LIHC; (d) Expression of 25 DEGs in LIRI-JP. (^∗^*P* < 0.05, ^∗∗^*P* < 0.01, and ^∗∗∗^*P* < 0.001).

We first compared the transcriptional expression of HOXs in 374 HCC and 50 noncancerous samples from TCGA-LIHC (Figure 2(a)). The result showed that the mRNA levels of HOXs were generally higher in HCC. Then, we analyzed the differences between 243 HCC and 202 noncancerous samples from the LIRI-JP cohort. Similar to our previous result, the mRNA levels of the HOX family members in HCC were generally higher than those in the noncancerous samples, except for HOXB4. A total number of 25 members (Figure 2(b)); HOXA3, 6-7, 9-11, 13, HOXB8-9, 13, HOXC5-6, 8-11, 13, HOXD1, 3-4, 8-11, and D13 were significantly upregulated (|log2 FC| > 1.5, *P* < 0.05) in the two cohorts (Figures 2(c) and 2(d)) (Table S1).

Altogether, the expression of HOX genes was generally increased in the HCC tissues, suggesting that they may play important roles in HCC.

### 3.2. Methylation Patterns of HOX Genes in HCC

**Figure 3:**
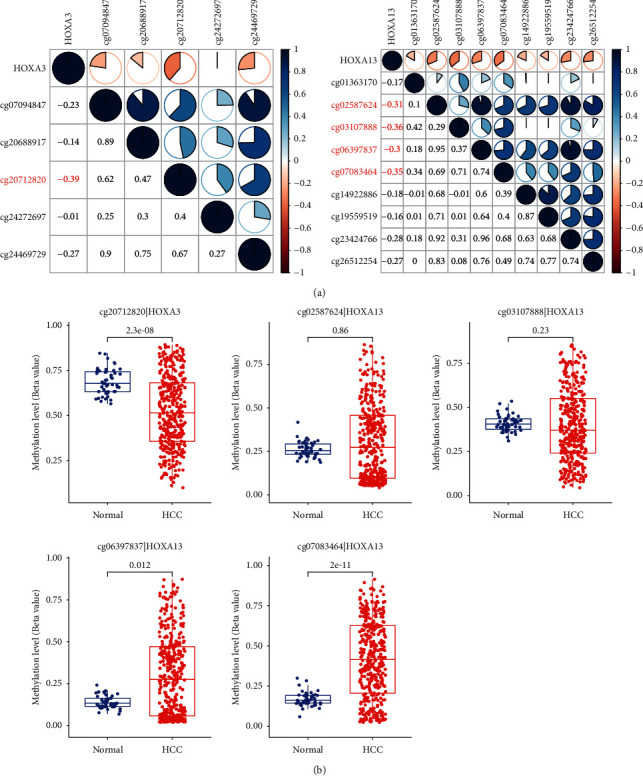
Methylation patterns of HOX genes in TCGA-LIHC. (a) Correlation between the methylation and expression of HOX genes. The methylation levels of 5 CpG sites were negatively correlated with the expression of the corresponding HOX gene (*r* < −0.3); (b) differences in the methylation levels of 5 CpG sites in HCC and noncancerous tissues. Three of the five CpG sites were differentially methylated positions between HCC and noncancerous tissues.

We first analyzed the correlation between the methylation levels of different CpG sites in the promoters of the HOX genes and the corresponding expression of the HOX genes based on the data of the TCGA-LIHC cohort (Figure S2). A total number of 5 CpG sites showed a significant negative correlation (*r* < −0.3) between the methylation level and the corresponding gene expression (Figure 3(a)). Then, we analyzed the differences in their methylation levels between HCC samples and noncancerous samples (Figure 3(b)). We found that the methylation levels of cg20712820 in HCC were significantly lower than those in the noncancerous samples. Conversely, the methylation levels of cg06397837 and cg07083464 in HCC were significantly higher than those in the adjacent noncancerous samples. These data suggested that these three CpG sites may be closely associated with HCC.

### 3.3. Diagnostic Value of HOXs in HCC

**Figure 4:**
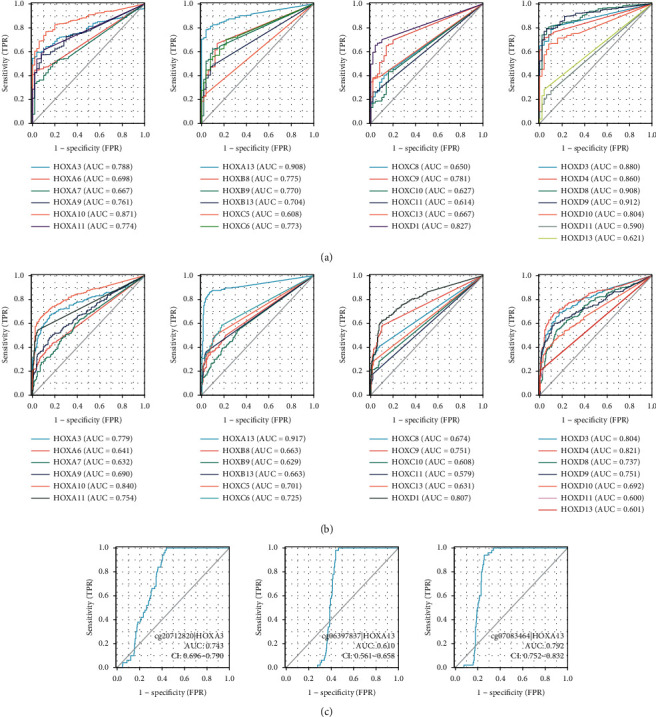
Diagnostic value of HOXs in HCC. ROC curves of HOX genes expression in TCGA-LIHC (a) and LIRI-JP (b); ROC curves of the methylation levels of three differentially methylated CpG sites in TCGA-LIHC (c). AUC: area under the curve.

First, the diagnostic value of HOX genes expression was assessed by performing ROC curve analysis based on the expression data collected from the TCGA and ICGC databases (Figures 4(a) and 4(b)). We established that a total number of five HOX members (HOXA10, 13, D1, 3, and D4) had superior predictive power (AUC >0.8) in both cohorts. Among them, the AUC of HOXA13 was higher than 0.9 (0.91 and 0.92, respectively).

Next, we also assessed the diagnostic value of three differentially methylated CpG sites (Figure 4(c)). We detected a correlation between the methylation of cg20712820 and cg07083464 and HCC incidence (AUCs of 0.74 and 0.79, correspondingly).

The above data suggested that the expression of HOXA10, 13, D1, 3, and D4 could serve as potential markers for the diagnosis of HCC, especially HOXA13. Besides, the methylation levels of cg20712820_HOXA3 and cg07083464_HOXA13 also had moderate value for the identification of HCC.

### 3.4. Prognostic Value of HOXs in HCC

**Figure 5:**
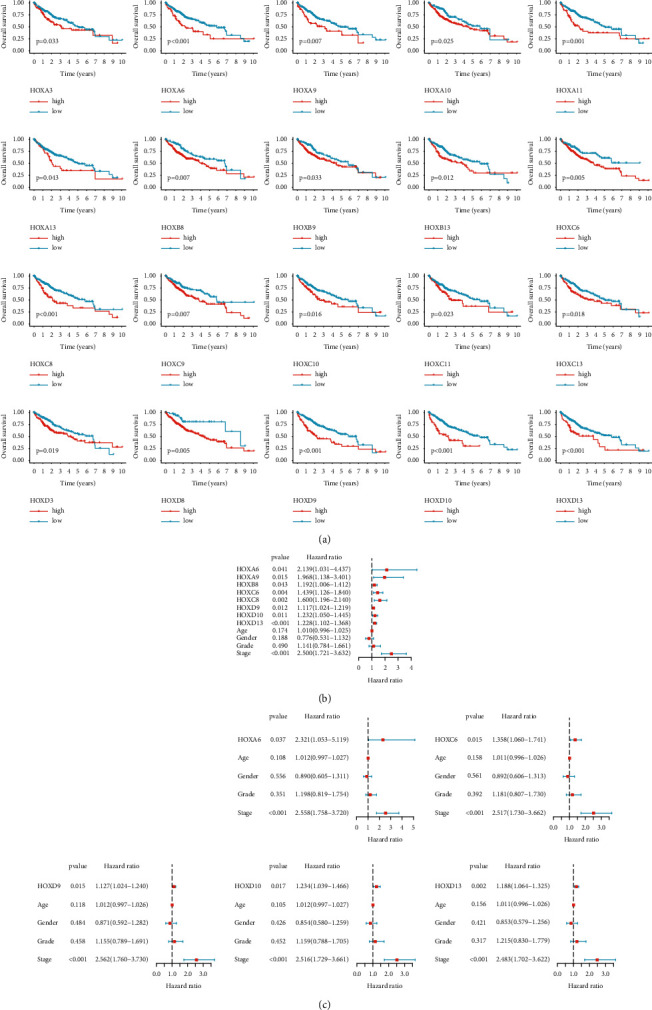
Prognostic value of HOXs in HCC. (a) K–M analysis for OS of patients stratified by HOX genes expression; (b) univariate Cox analysis; (c) multivariate Cox analysis of HOX family genes and clinical factors.

The clinical characteristics of all HCC patients included in our survival analysis are displayed in Table 1. First, the prognostic value of the HOX family members was evaluated using the K–M method. As can be observed in Figure 5(a), the high expression of HOXA3, 6, 9-11, 13, B8-9, 13, C6, 8-11, 13, D3, 8-10, and D13 was related to poor OS. Moreover, the results of the Cox regression model revealed that the elevated expression of HOXA6, 9, B8, C6, 8, D9-10, and D13 was significantly associated with unfavorable OS (Figure 5(b)). Five of them (HOXA6, C6, D9-10, and D13) were independent risk factors (Figure 5(c)). These results indicated that HOXA6, C6, D9-10, and D13 could serve as markers for predicting the prognosis of HCC patients.

### 3.5. Genetic Alterations of HOXs in HCC Patients

**Figure 6:**
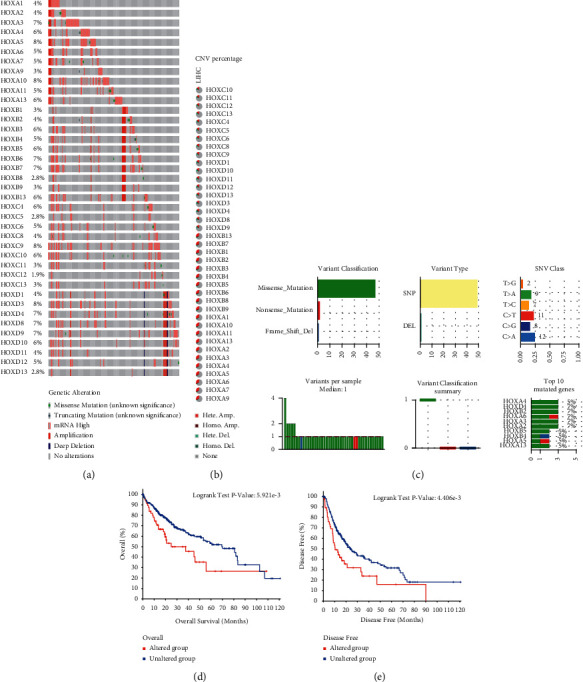
Genetic alterations of HOXs in TCGA-LIHC. (a) The genetic alteration rate of HOXs; (b) the constitute of CNV of HOXs; (c) the details of SNV of HOXs; (d, e) Differences in OS and DFS between patients with CNV or SNV and patients without CNV or SNV.

To further explore the role of HOX family in HCC patients, we assessed the genetic alterations of the HOX members. We first analyzed the mutation, CNA, and the expression data of TCGA-LIHC using the cBioPortal. The genetic alterations percentages of HOXs in HCC varied from 1.9% to 8% (Figure 6(a)). We next obtained the details of CNV and the mutations of HOXs from the GSCA database. We established that heterozygous amplification was the main type of CNV (Figure 6(b)), and missense mutations constituted the largest proportion of the mutations (Figure 6(c)). Moreover, the results of the K–M analysis obtained by using the cBioPortal showed poor OS and DFS in cases with mutations and CNV of HOX genes (Figures 6(d) and 6(e)).

In conclusion, the genetic alterations of HOXs in HCC patients were also associated with poor prognosis.

### 3.6. Functional Enrichment Analysis of Prognosis-Related HOXs

**Figure 7:**
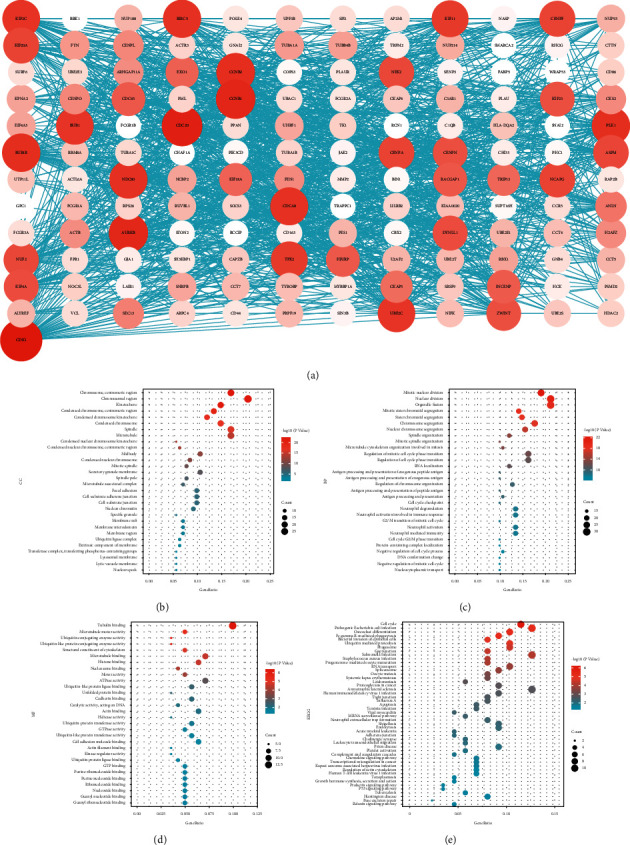
Functional analysis of the five prognosis-related HOXs. (a) PPI network of hub genes in coexpression network; (b) cellular components; (c) biological process; (d) molecular function; (e) KEGG pathways.

To further explore the mechanism by which the five HOXs influence HCC patients' prognosis, we first obtained the genes that were significantly correlated (|*r*| ≥ 0.4) with these HOXs in TCGA-LIHC from the UALCAN database. Then, STRING analysis was conducted to construct a PPI network, and Cytoscape was applied to screen the hub genes. As can be seen in Figure 7(a), there were 145 genes in the core network. The gene (CDK1) with the darkest red color and the largest node size had the highest degree in the network. Then, GO and KEGG enrichment analysis were performed to understand the potential function of the hub genes (Table S2, S3). The top 30 enriched categories of each GO group are depicted in Figures 7(b)–7(d). The KEGG pathways are illustrated in Figure 7(e). These results revealed that the hub genes were involved mainly in cell proliferation, cell cycle regulation, and immune response.

### 3.7. Relationship between the Five Prognosis-Related HOX Genes and the Tumor Microenvironment

**Figure 8:**
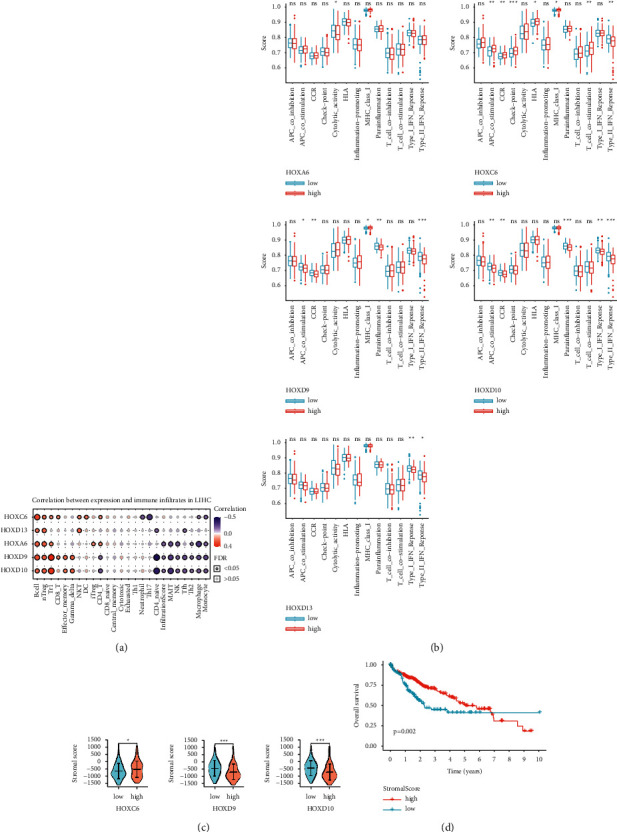
Relationship between the five prognosis-related HOX genes and the tumor microenvironment. (a) Correlation between HOXs expression and the abundance of immune cells. The bubble size correlates with FDR; (b) differences in the ssGSEA scores of the immune functions between the tissues with different expression levels of HOXs; (c) differences in the stromal scores between the tissues with different expression levels of HOXs; (d) K–M result for the OS of the patients stratified by the stromal score. (ns: no statistical significance, ^∗^*P* < 0.05, ^∗∗^*P* < 0.01, and ^∗∗∗^*P* < 0.001).

To further explore the roles of the five HOXs in tumor microenvironment (TME), we first used the GSCA database to analyze the correlation between the expression levels of these HOXs and the estimated abundance of 24 immune cell types (Figure 8(a)). We found that the five HOXs were associated with a number of immune cell types. Then we used the ssGSEA algorithm to analyze the effect of the five HOX genes on immune functions (Figure 8(b)). All HCC samples were divided into two groups based on the median HOXs expression level. The HCC samples with high HOXA6 expression had lower scores of their cytolytic activity. In contrast, the samples with high HOXC6 expression showed higher scores in multiple immune functions, such as check point, but obtained lower scores in the type II IFN response. Meanwhile, the scores of CCR, APC costimulation, and parainflammation of the groups with high expression of HOXD9 and HOXD10 were lower than those in the groups with low expression of these two HOXs. The samples with high expression of HOXD10 and HOXD13 had lower scores in both IFN response types. In addition, the higher expression of HOXD9 was also associated with a lower score of type II IFN response but a higher score of MHC class I.

Further, using the ESTIMATE algorithm, we also explored the association of the five HOXs with stromal cells, another important component of TME. As visible in Figure 8(c), the stromal scores in the tissues with high HOXC6 expression were higher than those in the tissues with low HOXC6 expression. However, the results of HOXD9 and HOXD10 were opposite to those of HOXC6. The further KM analysis showed that the patients with higher stromal scores had better overall survival (Figure 8(d)).

Taken together, the role of the five prognosis-related HOXs in HCC may be achieved in part by influencing the compositions and functions of TME.

### 3.8. Relationship between HOXC6 and Immune-Checkpoint Inhibitor Therapy

**Figure 9:**
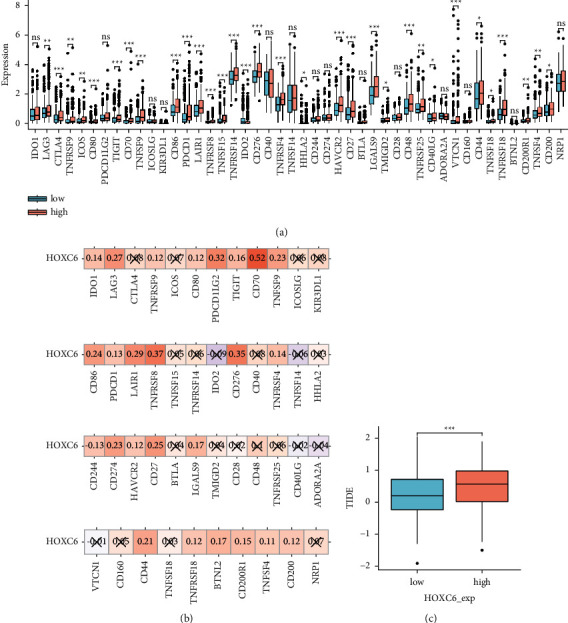
Relationship between HOXC6 and ICIs therapy. (a) Differences in the immune-checkpoint genes expression between HCC tissues with different expression levels of HOXC6; (b) correlation between the HOXC6 expression and the immune-checkpoint genes expression in TCGA-LIHC. The cross mark indicates *P* ≥ 0.05; (c) differences in the TIDE score between the tissues with different expression levels of HOXC6 (ns: no statistical significance, ^∗^*P* < 0.05, ^∗∗^*P* < 0.01, and ^∗∗∗^*P* < 0.001).

To elucidate the relationship between HOXC6 and immune checkpoint, we first investigated the expression differences of 46 immune-checkpoint genes between tissues with low and high HOXC6 expression. As can be seen in Figure 9(a), the median expression level of most immune-checkpoint genes was higher in the tissues with high HOXC6 expression than in those with low. Then, we measured the correlation between the expression of HOXC6 and immune checkpoints (Figure 9(b)). Our results evidenced that the expression of 25 genes was correlated with HOXC6 expression. Among them, PDCD1LG2 (*r* = 0.32), CD70 (*r* = 0.52), TNFRSF8 (*r* = 0.37), and CD276 (*r* = 0.35) were closely correlated with the expression level of HOXC6. It should be noted that the expression of PD-1 (PDCD1) had little correlation (*r* = 0.13) with the expression of HOXC6, and CTLA4 expression was independent of HOXC6 expression. Meanwhile, the Pearson correlation coefficient between PD-L1 (CD274) expression and HOXC6 expression was 0.23.

Then, TIDE algorithm was employed to predict the clinical response to anti-PD1 and anti-CTLA4 treatments. The TIDE score in HCC samples with high HOXC6 expression was higher than that in tissues with low HOXC6 expression (Figure 9(c)). However, HOXC6 expression was independent of TMB.

In conclusion, the high expression of HOXC6 in HCC might suggest poor outcome of anti-PD1 and anti-CTLA4 therapy. However, some other checkpoint genes, such as CD70, were associated with HOXC6, suggesting that HOXC6 might be a potential marker for therapy targeting these immune checkpoints in HCC.

### 3.9. Correlation between HOXs and Anticancer Drug Sensitivity

**Figure 10:**
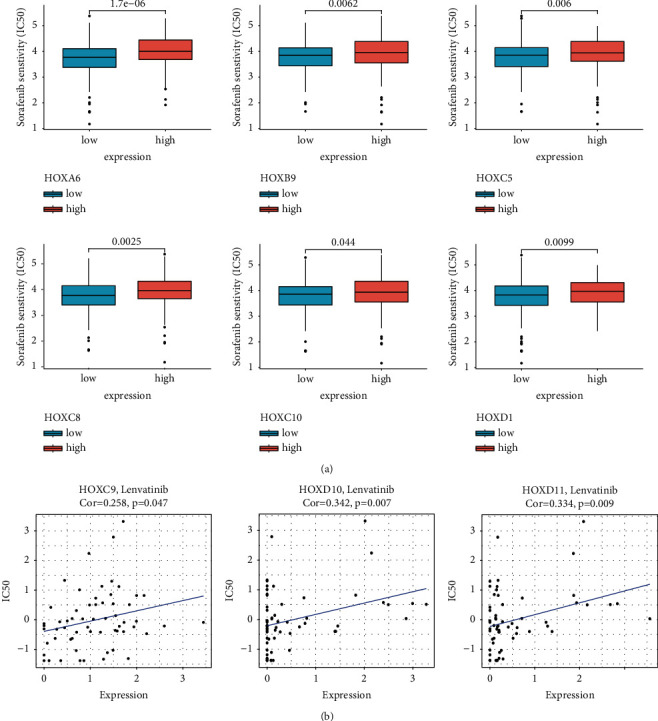
Relationship between the HOXs expression and anticancer drug sensitivity. (a) Differences in the estimated IC50 of sorafenib between HCC tissues with different expression levels of HOXs; (b) correlation between the HOXs expression and IC50 of lenvatinib in NCI-60 cancerous cell lines.

In the past decade, sorafenib has been the only systemic agent with proven clinical efficacy for patients with advanced HCC [35]. We first compared the estimated IC50 of sorafenib in tissues with low and high expression of HOX genes. As shown in Figure 10(a), tissues with high HOXA6, B9, C5, 8, 10, and D1 expression were less sensitive to sorafenib.

Then, the NCI-60 cancerous cell lines were used to measure the correlation between HOXs expression and the sensitivity to 218 FDA approved anticancer drugs. The expression of HOXC9, D10, and D11 were positively correlated with the IC50 of lenvatinib (Figure 10(b)), which was the first new drug approved for advanced stage HCC in the first-line setting in over 10 years [36]. Meanwhile, there were also significant correlations between the IC50 values of many drugs and the expression of HOX genes (Table S4).

Taken together, the expression of HOXs may be associated with the efficacy of many anticancer drugs, which might be another factor affecting the prognosis of cancer patients.

## 4. Discussion

The HOX genes were discovered in the human body at the end of the 20^th^ century, and have attracted widespread attention since then [8, 9]. Apart from their well-known roles in embryogenesis, for over 20 years, the links between HOX genes and human cancer have been comprehensively investigated. Accumulating evidence has shown the role of HOXs in many cancers [12–15]. However, the significance of most HOX members in HCC has remained unclear. To address this scientific gap, we conducted the present study, which is the first to comprehensively analyze the role of HOXs in HCC using multiple bioinformatics algorithms. We found that the increased mRNA levels of HOX genes in HCC were associated with poor prognosis. Among them, HOXA6, C6, D9, D10, and D13 were identified as independent risk factors. Functional analysis suggested that cell proliferation, cell cycle, and microenvironment regulation might be the main mechanisms of the involvement of these five HOXs in HCC development. Meanwhile, multiple HOX members (such as A13) showed excellent diagnostic value in HCC.

A previous study showed that the transcription of HOXs was silent in adult noncancerous liver tissues, whereas the expression levels of most HOXs in HCC were increased [37]. Moreover, in almost all HCC samples analyzed in another study, the mRNA content of HOXA13 in HCC tissues was over 100 times higher than that in normal liver tissues, strongly suggesting that HOXA13 was closely related to HCC [38]. In our study, the HOXA13 expression fold change was 191 in TCGA cohort and 161 in ICGC cohort after outliers' removal. Our results on the expression of HOX genes in HCC were consistent with those of previous studies. Abnormal methylation of HOX genes was also evidenced to be characteristic for some human cancers [16]. However, the published reports on the significance of methylation of HOXs in HCC are scarce. The hypothesis that HOXD3 was upregulated in HCC by methylation modification was proposed [39]. Here, we identified five CpG sites that might regulate the expression of corresponding HOX genes. Among them, the hypomethylation of cg20712820_HOXA3 and the hypermethylation of cg07083464_HOXA13 were closely related to HCC. Therefore, further research of these two CpG sites may be conducive to better understanding the role of HOXs in HCC.

Several studies have elucidated the clinical significance of these five HOXs in some cancers. HOXA6 was found to be associated with the proliferation, apoptosis, migration and invasion of CRC [40]. In ccRCC, HOXA6 inhibited cell proliferation and induced cell apoptosis by the suppression of the PI3K/AKT signaling pathway [41]. Our present results also suggested that HOXA6 may affect the proliferation and apoptosis of HCC. In an earlier investigation, the increased HOXC6 expression promoted the proliferation of HCC and reduced the sensitivity to 5-FU [42]. Meanwhile, HOXC6 promoted the invasion of HCC cells by driving EMT [43]. In addition, HOXD9 enhanced EMT and cell metastasis in HCC by ZEB1 regulation [44]. The HOXD10/RHOC/UPAR/MMPs pathway is related to the migration and invasion of HCC [45]. The aforementioned *in vitro* experiments have evidenced that these HOXs are involved in the progression of HCC. We also confirmed the effect of HOXs on the clinical outcomes of HCC patients by analysis of large-sample follow-up data. Notably, the drug sensitivity data of the present study suggested that HOX genes may have guiding significance in the treatment of HCC and even pan-cancer.

The disturbance of various components of TME also contributes to the malignant features of HCC [46]. As one of the main components of TME, the abundance of immune cells, especially T cells, is closely associated with tumor progression [47]. Tregs are the major immunosuppressive and anti-inflammatory cells that can inhibit the T-cell response through IL-17 and IL-6 activities, leading to T-cell exhaustion and immune escape [48, 49]. NK cells were found to be the main antitumor cells in the liver [50]. NKT cells can directly kill tumor cells by recognizing the CD1d antigen or by activating NK cells, and the number of NKT cells is positively correlated with OS and RFS of HCC patients [51, 52]. Here, we also focused our attention on some crucial immune response processes. Cytolytic activity (CYT) reflects the ability of cytotoxic T cells and NK cells to lyse tumor cells [53]. A recent study found that higher CYT values in HCC indicate greater immunogenicity and more favorable TME, which leads to better prognosis [54]. This might be a mechanism by which HOXA6 expression is associated with poor prognosis. The absence of the costimulatory molecules renders tumors invisible to the immune system, whereas inhibitory molecules protect tumors from effective T cells [55]. Chemokines are the bridge between inflammation and tumor, and control several aspects of tumor biology, such as immune infiltration, angiogenesis, proliferation and migration [56]. IFN response plays crucial roles in promoting host antitumor immunity and is considered to be pivotal components in the cancer-elimination phase of the cancer immunosurveillance [57]. The expression of HLA is related to tumor immune escape, and it is considered to act as a tumor suppressor [58]. It can be inferred that these HOX genes may be regulators of TME that influence the patient's clinical outcome by their effects on antitumor immunity. Nevertheless, the mechanisms through which they shape the TME remains to be further explored.

Certain limitations of our study are to be acknowledged. First, we analyzed the expression of the HOX family genes only at the mRNA level. Thus, it is necessary to further investigate the role of HOXs at the protein level. Second, our results on the molecular mechanism of HOXs need to be verified by further experiments. We will focus on addressing these issues in future studies.

## 5. Conclusions

In conclusion, HOX genes expression was generally upregulated and correlated with poor prognosis in HCC. HOXA6, C6, D9-10, and D13 are independent risk factors that might affect patients' prognosis through multiple pathways. The transcription and methylation characteristics of HOXs also had excellent diagnostic efficacy. Therefore, the HOX family genes might play important roles in the occurrence and development of HCC and thus could be exploited as effective biomarkers for HCC diagnosis and prognosis.

## Figures and Tables

**Table 1:** Clinical characteristics of the HCC patients.

Characteristic	Type	*N*	Proportion (%)
Age	≤65	232	62.70
	>65	138	37.30

Gender	Male	249	67.30
	Female	121	32.70

Histologic grade	G1-2	232	62.70
	G3-4	133	35.95
	Unknown	5	1.35

Pathologic stage	Stage I-II	256	69.19
	Stage III-IV	90	24.32
	Unknown	24	6.49

Vital status	Dead	130	35.14
	Alive	240	64.86

## Data Availability

The data of this study were collected from the TCGA and ICGC databases.

## Supplementary Materials

Supplementary Materials Figure S1: genomic organization of human HOX genes. Figure S2: Pearson's correlation between the methylation and expression of HOX genes in TCGA-LIHC. Table S1: results of differential expression analysis of HOX genes. Table S2: results of the GO analysis. Table S3: results of the KEGG analysis. Table S4: Pearson's correlation between HOX genes expression and IC50 of anticancer drugs in NCI-60 cell lines.
