# Supplementary figures and images for: Comprehensive Analysis of HOX Family Members as Novel Diagnostic and Prognostic Markers for Hepatocellular Carcinoma
Source: J Oncol. 2022 Feb 23;2022:5758601. doi: 10.1155/2022/5758601 (PMC8890896; doi:10.1155/2022/5758601)

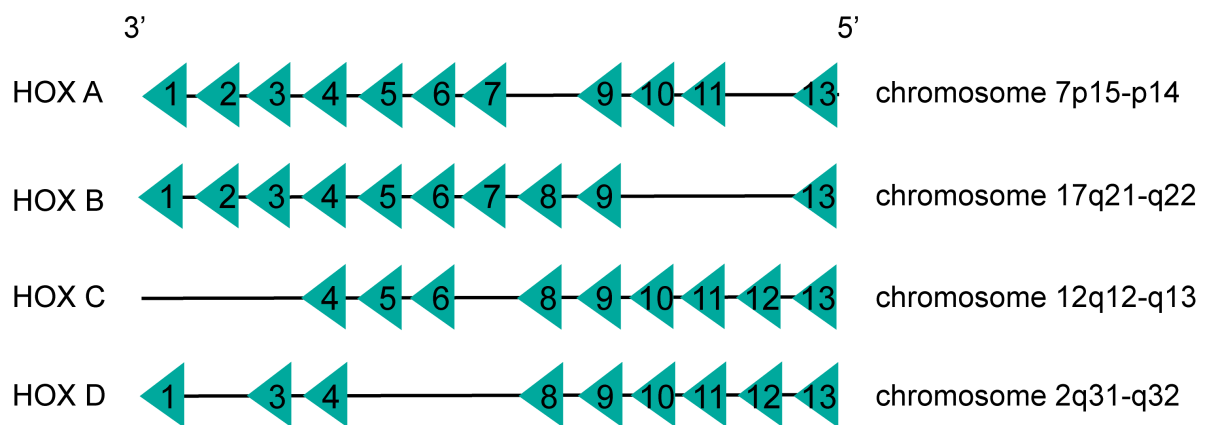

Supplement: Supplementary Materials — Figure S1: genomic organization of human HOX genes. Figure S2: Pearson's correlation between the methylation and expression of HOX genes in TCGA-LIHC. Table S1: results of differential expression analysis of HOX genes. Table S2: results of the GO analysis. Table S3: results of the KEGG analysis. Table S4: Pearson's correlation between HOX genes expression and IC50 of anticancer drugs in NCI-60 cell lines. [file 5758601.f1.zip › Supplementary Materials/Figure S1 Genomic organization of human HOX genes.pdf]

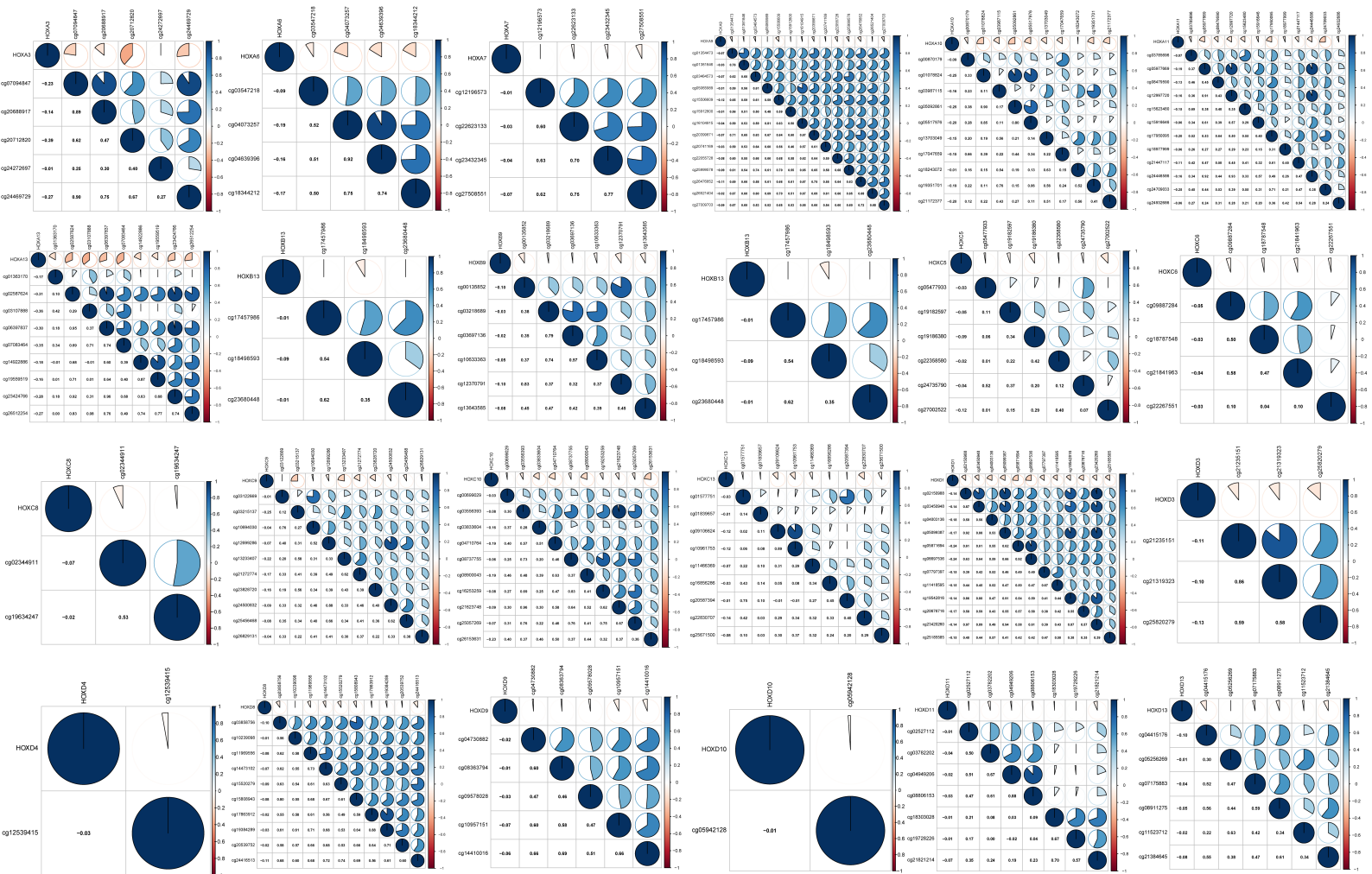

Supplement: Supplementary Materials — Figure S1: genomic organization of human HOX genes. Figure S2: Pearson's correlation between the methylation and expression of HOX genes in TCGA-LIHC. Table S1: results of differential expression analysis of HOX genes. Table S2: results of the GO analysis. Table S3: results of the KEGG analysis. Table S4: Pearson's correlation between HOX genes expression and IC50 of anticancer drugs in NCI-60 cell lines. [file 5758601.f1.zip › Supplementary Materials/Figure S2 Pearsoní»s Correlation between the methylation and expression of HOX genes in TCGA-LIHC.pdf]
